# Construction Notes

**Published:** 1894-08-25

**Authors:** 


### CONSTRUCTION NOTES.

### Wolverhampton Borough Hospital New Pavilion.

The new isolation pavilion has been formally inspected by the Sanitary Committee, and is now open for the reception of smallpox cases. Additional accommodation has been rendered necessary on account of the existing permanent buildings being at the present time fully occupied by scarlet fever patients. The new building is situate in an isolated site, and is efficiently drained and fitted with the latest sanitary appliances, lavatories, accommodation for baths, arrangements for cooking, &c., the wards being heated by slow combustion stoves. Gas is laid on throughout.

**Extension of Gravesend Hospital.**

A new wing, designed by Mr. N. C. H. Nisbett, A.R.I.B.A., has just been added to the above institution. Two large wards, 43 ft. by 25 ft., and two smaller ones provide for all the adult patients. The two wards on the ground floor are devoted to men, the upper ones to women. There is also a smaller special ward on each floor for special cases, and a lift of sufficient size to take a bed will communicate with the upper floor. A new kitchen and scullery and board-room have been provided, as also a matron's room. The out-patients have the whole of the present block facing Bath Street, the rooms on the south side being thrown together to form a waiting-room. The present consulting-room will be re-arranged, and the adjoining dispensary utilised as an examination room for ophthalmic and other special cases in conjunction with it. The dispensary will be removed to the front, and a room for patients waiting for medicine provided next to it. By this arrangement the out-patients will be entirely cut off from the rest of the hospital. The porter's lodge will be between the new entrance and existing building, with the secretary's office in close proximity. Considering more in detail the new wards, a special feature is the entire avoidance of any fittings, &c., which might harbour dirt. All internal angles of the walls with one another or with ceilings will be rounded. Ward sculleries are provided just outside the wards, with hot and cold water and a gas stove, for cooking small dishes and other requirements. The contractor for the building is Mr. L. Seager, of Sittingbourne, and the amount of the contract is under £4,700.

